# Supplementary material for: Are carbon emissions trading and green financial instruments synergistic? -Comprehensive quantitative research based on content analysis
Source: PLoS One. 2024 Mar 7;19(3):e0298601. doi: 10.1371/journal.pone.0298601 (PMC10919601; doi:10.1371/journal.pone.0298601)
Supplement: S1 Data — (ZIP) [file pone.0298601.s001.zip › Data/地级市Z_F工作报告.zip!/╕╩╦α/┬ñ─╧╩╨/└┤╫╘╣1⁄2╓┌║┼í╛┬φ┐╦╩2╛▌═°í┐_2010.docx]

各位代表：

现在，我代表市人民政府向大会作政府工作报告，请予审议，并请各位政协委员和列席人员提出意见。

2009年工作回顾

刚刚过去的2009年是我市困难较多、建设任务十分繁重的一年。面对灾后恢复重建的艰巨任务、金融危机的严重冲击和自然灾害的影响，我们以科学发展观为指导，在省委、省政府的坚强领导下，紧紧围绕贯彻落实市委的决策部署和市二届四次人代会确定的目标任务，坚持一手抓灾后恢复重建，一手抓经济社会发展，全力推动保增长、保民生、保稳定各项工作，灾后恢复重建取得新进展，经济社会保持平稳较快发展，基本完成了各项目标任务。全年完成生产总值130亿元，同比增长9%；完成全社会固定资产投资200亿元，同比增长69%；完成大口径财政收入17亿元、市县级财政收入7.9亿元，同比分别增长2.2%、3.5%；完成社会消费品零售总额35亿元，同比增长16.5%；城镇居民人均可支配收入9477元，同比增长9%；农民人均纯收入1995元，同比增长12.1%（若含救灾补助达到2300元，同比增长15.1%）。

----灾后恢复重建取得重要阶段性成果。我们将灾后恢复重建作为政府工作的重中之重，按照“三年重建任务力争两年基本完成”的总体要求，加强组织领导，调整完善实施计划，着力破解重建难题，加强项目前期工作，简化项目审批程序，多方争取重建资金，协调落实农民住房重建贷款，加大农村贫困户建房帮扶力度，强化资金和建材市场监管，扎实有序地推进灾后恢复重建。累计开工重建项目1793项（不含城乡住房），竣工462项，累计到位资金207.6亿元，累计完成投资197亿元（含城乡住房）。农村居民住房维修加固186459户全部竣工，农村居民住房重建227932户基本完成，农村住房条件明显改善。城镇居民住房维修加固和重建全面启动实施；县以下学校、医院重建进度较快。基础设施等重建项目取得明显进展。深圳市直接实施援建项目19项，已竣工13项，在建6项，累计完成投资3.92亿元。省内对口援建工作顺利开展。

----项目建设和招商引资取得新成效。我们认真研究解决兰渝铁路、武罐高速公路、阳山金矿等重大项目建设征地拆迁、供水供电等具体问题，保证了项目顺利实施，兰渝铁路(陇南段)完成投资16.7亿元；武罐高速公路23个标段全部完成招投标，其中国内投资的主线10个标段开工建设，完成投资2.11亿元。争取扩大内需项目293项，投资8.65亿元。开工建设投资50万元以上的项目1028项，当年完成投资106亿元。全市签约招商引资合同项目180个，拟引进资金75亿元，到位资金40亿元，合同履约率达到80%。去年，全市项目总量和全社会固定资产投资均创历史最好水平。

----农业和农村经济持续发展。我们突出“三农”工作的基础地位，认真落实惠农强农政策，发放各类补贴7.61亿元；大力实施农民增收行动计划，狠抓劳务输转，全年完成农村劳动力技能培训14.5万人，输转劳动力65.9万人，创收43.5亿元；加大对特色农业产业的支持力度，市财政筹措1000万元发展基金支持特色农业产业开发，核桃、花椒、油橄榄、中药材、蔬菜等面积均有增加，肉蛋奶总产量达到15.6万吨，改扩建龙头企业19户。成功举办了全国第六届干果生产与科研进展学术研讨会暨中国陇南特色果业高层论坛。加强了农业基础设施建设，新增有效灌溉面积3.7万亩、节水灌溉面积4万亩、补灌面积4万亩，实施天保工程天然林管护1013万亩，治理水土流失410平方公里，新修梯田13.98万亩，提高了农业抗御自然灾害的能力和综合生产能力；完成全膜双垄沟播20.7万亩，粮食总产达到96.61万吨。

----工业经济企稳回升。面对工业企业生产经营困难、工业经济下滑的严峻局面，我们把保工业作为保增长、保就业的重点，认真落实市委二届六次全委扩大会议的决策部署和《关于进一步加快工业发展的意见》，加大财税对工业的支持力度，市级财政安排1000万元工业发展专项资金、200万元工业技改贴息资金，并从企业所得税收益中列支10 %以上用于扶持工业发展。召开银企合作洽谈会，协调解决中小企业融资难问题。狠抓工业项目建设，市列40个工业重点项目开工36项，完成投资17.9亿元，武都100万吨新型干法水泥生产线建成投产，成县120万吨新型干法水泥、成州公司10万吨锌冶炼技改、康县独一味三期扩建等重点工业项目进展顺利。强化协调服务和政策扶持，铅锌采选企业全部恢复生产，工业经济逐月回升。完成工业增加值23.14亿元，同比增长2.4%；其中规模以上工业完成增加值17.14亿元，同比增长2.3%。节能减排任务全面完成，二氧化硫排放总量和化学需氧量分别控制在0.88万吨和0.87万吨之内，全市万元GDP综合能耗下降5.1%。产品质量安全和监督工作得到加强。

----城乡基础设施不断改善。我们把加快基础设施建设、解决瓶颈制约作为重要抓手，开工建设国省干线公路灾后恢复重建项目10项1083公里，完成投资13.8亿元。实施农村公路建设项目1340项8225公里，完成1083项5918公里，其中通乡油路28项715公里，通村公路1055项5203公里。召开了全市城市建设现场会，强化城市建设管理工作，《陇南市城市总体规划》通过省级专家评审；实施城镇建设实事122件，已完成54件，正在实施68件；市区东江、钟楼滩新区开发建设和八县县城建设取得新进展。武都、西和330千伏送变电工程建成投运。新建光缆线路1360公里，九县区城区和重点乡镇实现了3G信号覆盖。

----第三产业较快发展。我们把发展第三产业、搞活农村市场作为拉动内需的重要措施，开展“家电下乡”、“汽车以旧换新”活动，销售家电21752台（部）。推进“万村千乡”市场工程，实施市场建设项目53个，新建农家店380个，市场体系建设明显加快。完成了粮食企业改革并实现扭亏为盈，供销企业持续保持盈利态势。积极推进旅游产业开发，加快景区基础设施建设，加强旅游宣传促销，全年接待游客比上年增长25.6%，旅游综合收入增长45.3%。非公有制经济较快发展，全市中小企业实现增加值38.9亿元，同比增长8.2%。金融机构存贷款快速增长，全市存款余额341亿元，同比增长17.9%；贷款余额148亿元，同比增长48%。

----社会事业全面发展。我们多方争取项目资金，实施了一批社会事业基础设施建设项目，社会事业发展的基础条件明显改善。坚持优先发展教育，中小学校舍安全工程启动实施，宕昌县“两基”攻坚通过省上验收，全市整体实现了“两基”目标。完成科技培训51万人次，取得科技成果20项，专利授权20件。卫生基础设施建设项目扎实推进，甲型H1N1流感、手足口病等传染病防控工作有效进行，食品药品监管力度进一步加大。建成文化大院6家、农家书屋640家。八县一区城区有线电视数字化转换基本完成。人口低生育水平保持稳定，全市人口出生率控制在14.2‰以下，农村计划生育率达到88%以上，完成了省政府下达的计划目标。全民健身活动广泛开展。促进城乡就业的优惠政策得到落实，城镇登记失业率控制在3.8%以内。解决了9831名村干部养老保险和5619名关闭破产国有企业退休职工医疗保障问题，年末参加基本养老保险、失业保险、医疗保险、工伤保险的人数均有较大增长，城镇居民基本医疗保险参保率、农民参加新型合作医疗率分别达到93%和95.78%，农民工参加工伤保险取得新的突破，各项保险资金按时足额拨付。全市城镇职工基本医疗、失业、工伤保险和城镇居民医疗保险开始由县级统筹向市级统筹过渡，社会保险保障层次和水平进一步提高。认真落实民族政策，少数民族乡村经济社会事业得到发展。采取积极措施应对暴洪等自然灾害，加强地质灾害防治和气象预测预报，全力做好应急救援和困难救助工作，受灾和困难群众的生产生活得到妥善安置。解决了8000多名残疾人的生活困难。安全生产形势总体稳定，事故起数、死亡人数、受伤人数、经济损失同比分别下降11.8%、27.8%、21.1%、18.7%。深入推进社会治安综合治理，调处各类矛盾纠纷6100多件，集中解决了一批信访积案，维护了社会大局稳定。双拥共建不断深化，部队建设迈出新步伐。农村综合改革、政府机构、事业单位人事制度、医药卫生、财政金融、教育体制等各项改革有序推进。统计、物价、老龄、档案、外事侨务等工作取得了新进展。

----改善民生办实事取得新成效。市委、市政府确定的十二件实事基本完成。投资2.34亿元，在551个灾后重建重点村配套建设供水管网1261公里、供电线路501公里、通村公路1005公里、堤防63公里。投资1.14亿元，建成便民桥113座。投资2.12亿元，建成通村等级公路464条2043公里。投资7566万元，解决了11万农村人口饮水不安全问题。投资8538万元，实施扶贫整村推进项目110个，减少贫困人口7.6万人。建成农村户用沼气池5.21万户；为农民配备太阳灶4.6万台。投资3.21亿元，开工建设廉租住房项目19项13万平方米、经济适用住房项目18项13.96万平方米，发放住房补贴和核减租金513万元，解决了5087户城市低收入家庭住房困难。从2009年1月起将城市低保标准提高了10%，农村低保补差由年人均516元提高到600元，农村五保供养标准由1200元提高到1600元。争取中央彩票公益金382.3万元，资助九县区贫困高中生3823人，每人资助1000元。结合实施农民住房灾后恢复重建，建成“五保家园”554个。建成了八县一区10个城区社区卫生服务中心。为65个城镇社区落实工作补助经费221万元。

----民主法制建设不断加强。我们把民主法制建设作为推动发展的重要保障，修订了政府工作规则，进一步完善了工作程序，政府工作科学化、规范化水平进一步提高。开展了行政执法人员学习培训、执法主体单位、执法人员资格确认工作。行政审批制度改革进一步深化，先后清理出审批、核准等事项588项，向社会公布了两批取消、下放、转移的审批等事项318项。认真落实党风廉政建设责任制，惩治和预防腐败体系建设稳步推进，灾后重建及其它项目全部按规定实行了公开招标投标；启动实施了灾后重建资金物资监管预防腐败试点工作。进一步加强审计监督，完成审计任务439项，查出各类违规资金9887万元。办理人大代表建议和政协委员提案515件，办复率达到100%。坚持优化发展环境，机关工作作风得到转变，服务发展、服务项目建设的意识进一步增强。

各位代表，过去的一年，我市灾后恢复重建和经济社会发展取得的成绩来之不易，这些成绩的取得，得益于省委、省政府的正确领导、关怀支持，省委、省政府对我市灾后恢复重建和经济社会发展给予了前所未有的关心和支持，极大地激发了我们战胜困难、加快发展的决心和信心；得益于市委的坚强领导、科学决策，市委总揽全局，科学判断形势，深化市情认识，提出的加快陇南科学发展的总体思路和重大措施，为我们谋划发展、推动发展提供了重要指导；得益于人大、政协和社会各界人士的监督支持，人大、政协和社会各界人士寓支持于监督之中，为促进灾后恢复重建、经济社会发展、社会和谐稳定倾注了心血，贡献了力量，发挥了不可替代的重要作用；得益于各县区、各部门的扎实工作、共同努力，各县区、各部门认真贯彻落实市委、市政府的决策部署，创造性地开展工作，形成了心齐劲足的良好局面；得益于深圳市、省内兄弟市和社会各方面的无私援助、倾力帮扶，特别是深圳市对我们给予了多方面的帮助和支持；得益于全市各族人民的艰苦奋斗、无私奉献，面对严酷条件和重重困难不屈服、不退缩，依靠自己勤劳的双手重建家园，恢复发展生产，凝聚了加快发展的力量，成为我们攻坚克难的强大动力。在这里，我代表市政府，向奋战在各条战线上的劳动者，向驻陇南人民解放军、武警官兵和中央、省驻陇南单位，向各民主党派、工商联、无党派爱国人士、离退休老干部，向所有关心支持陇南经济发展、社会进步的各界人士表示衷心的感谢和崇高的敬意！

各位代表，过去的一年，我们抢抓机遇，全力应对困难和挑战，付出了艰辛的努力，在历届班子和广大群众奠定的基础上推动了陇南的发展。但我们也清醒地认识到，全市经济社会发展还面临不少突出矛盾和困难。灾后恢复重建资金缺口比较大，农村异地重建村水、电、路等配套设施建设任务仍很繁重，农村受灾群众后续生活困难，重建项目实施特别是城镇居民住房重建进展比较缓慢；金融危机影响仍然存在，经济回升的基础尚不稳固，工业经济运行、财政增收仍面临较多困难；项目建设环境仍不宽松，项目管理工作仍比较薄弱，项目实施水平不高；社会事业发展仍显滞后，就业和再就业压力仍然很大，贫困人口较多，改善民生的任务仍很艰巨；随着重大项目建设和灾后重建的推进，各类利益矛盾不断出现，影响社会稳定的因素增多；政府工作还需要进一步加强和改进，少数公务人员还存在工作责任性和服务意识不强、工作落实不力、办事效率不高、为政不廉等问题，发展环境还需要进一步优化，工作作风、工作效率、工作水平还需要进一步改进提高。对此，我们将在今后的工作中努力加以解决。

2010年全市经济社会发展的主要任务和工作重点

2010年是全面完成灾后恢复重建任务的关键之年，也是贯彻实施省委、省政府“中心带动，两翼齐飞，组团发展，整体推进”区域发展战略，完成“十一五”规划、谋划“十二五”发展的重要一年，做好今年的工作具有特殊重要的意义。纵观发展形势和环境条件，我们面临的发展机遇和有利因素大于困难和挑战。从外部环境看，国际国内经济形势整体向好；国家继续实施扩大内需的政策措施，继续实施积极的财政政策和适度宽松的货币政策，将进一步加大对西部地区和贫困地区的支持力度；省委、省政府实施新的区域发展战略，一系列改革开放发展措施为我市的发展提供了新的机遇。从市内看，今后一个时期，还将有一批灾后重建和交通基础设施等重大项目相继实施，投资总量将进一步增加，基础条件将进一步改善，工业经济将进一步回升，城乡消费将进一步趋旺。特别是市委提出的发展思路得到了全市上下的一致认同，对经济社会发展的推动作用将更加显现出来。我们要坚定信心，迎难而上，加快发展。

根据全国、全省经济工作会议精神和市委的总体部署，今年全市经济社会发展的总体要求是：全面贯彻落实市委提出的“以科学发展观为指导，以富民强市为目标，围绕发展与和谐两大主题，抢抓灾后重建、扩大内需、对口援建三大机遇，实现项目带动、交通建设、城市建设三大突破，突出特色农业、工业发展、旅游开发、科教兴市、改善民生五个重点，强化加强党的建设、创新体制机制、加强资源整合、优化发展环境四项保证，促进全市经济社会又好又快发展”的总体思路，以项目建设为重点，以转变经济发展方式和优化经济结构为主线，扎实推进灾后恢复重建，着力培育主导产业，全力改善基础设施，更加注重改善民生，力争全面完成“十一五”规划任务，科学谋划和编制“十二五”规划。

综合分析发展的基础和潜力，今年全市经济社会发展的主要目标是：生产总值增长10%，达到146亿元；全社会固定资产投资增长20%，达到240亿元；大口径财政收入增长5%，达到17.9亿元，市县级财政收入增长3%，达到8.1亿元；工业增加值增长18%，达到27.3亿元，其中规模以上工业增加值增长16%，达到21亿元；社会消费品零售总额增长18%，达到41.36亿元；城镇居民人均可支配收入增长9%，达到10330元；粮食总产量达到96.61万吨以上；农民人均纯收入增长12%，达到2234元（不含灾后重建因素）；居民消费价格总水平涨幅控制在3%左右；城镇登记失业率控制在3.9%以内；人口自然增长率控制在6.8‰以内；万元生产总值能耗下降4.2%。

为实现上述目标，我们将本着统筹兼顾、突出重点的原则，着力抓好以下十个方面的工作：

一、切实采取有力有效措施，全面完成灾后重建任务。要切实把灾后恢复重建作为工作的重中之重，进一步细化目标任务，靠实工作责任，加强工作力量，强化工作措施，全面完成灾后恢复重建各项任务。一是抓好农民住房重建完善提高和验收工作。在抓好农民住房重建扫尾工作的同时，加强整村重建村水、电、路等配套基础设施建设。把住房建设、配套基础设施建设与产业发展、农民增收结合起来，制定产业发展规划，实施增收项目，搞好灾区群众后续生活扶持和产业发展，多渠道增加群众收入。善始善终做好农民住房重建验收工作。积极争取启动实施灾区移民工作，力争完成移民工作任务。二是加快城镇居民住房重建。坚持因地制宜，以原址重建住房、置换土地重建住房、租赁住房、购买安居住房、单位组织重建住房、置换安居住房、一次性货币终结、购置商品房等不同类型的重建模式开展重建工作。建立领导干部包社区、包项目责任制，加大工作力度，改进薄弱环节，落实国家、省上出台的财政扶持、税费减免、信贷优惠和土地供给等优惠政策，严格落实廉租住房、经济适用房配建政策，整合项目资金，加快推进城镇居民住房灾后恢复重建。三是加快重建项目进度。严格实施灾后恢复重建规划，全面加快公共服务、城镇体系、农村建设、基础设施建设、生产力布局和产业调整、市场服务体系、防灾减灾、生态环境恢复等重建项目建设，加强项目设计、审批、征地等前期工作，确保各类重建项目如期竣工。整合行政、事业单位办公和业务用房建设项目，加快机关单位重建步伐。四是继续加强对口援建工作。进一步完善对口支援协调机制，做好协调服务工作，为援建方创造良好的建设环境，全面实施好援建项目。构建对口支援长效机制，深化与深圳市、省内援建市和援建单位、企业在资源开发、产业转移、工业园区建设、干部交流、人才培养、劳务输出、特色农产品销售等方面的交流合作，力争取得新成效。五是保证灾后恢复重建质量。强化项目管理，严格执行项目法人责任制、招标投标制、工程质量监理制、合同管理制及工程质量责任追究制。严格执行抗震设防技术规程和国家建筑技术规范，确保重建工程质量。六是加强资金和建材市场监管。完善资金拨付制度，健全管理台帐，加强资金使用监管，严肃查处各类违法违纪案件，保证重建资金安全，发挥重建资金的最大效益。继续抓好建材生产、调运，保障建材供应，确保建材质量，确保建材价格基本稳定。立足当前，着眼长远，积极争取灾后重建后续政策扶持。

二、着力解决制约因素，加快推进项目建设。按照基础设施和社会事业项目靠争取，产业发展和城市建设项目靠招商的思路，下决心解决项目建设环境不宽松、人才缺乏、前期工作滞后、用地落实困难、管理工作薄弱、实施进度缓慢、后续项目储备不足等突出问题，意向性项目抓落地、前期项目抓开工、开工项目抓投产、投产项目抓效益，力争在项目论证、储备、争取、实施、管理上有新突破。一是加快重点项目建设。认真做好项目土地征用、房屋拆迁、审批办证等服务工作，为兰渝铁路、武罐高速公路、阳山金矿等重大项目建设搞好服务，营造良好的项目建设环境，保证项目顺利实施。二是切实抓好扩大内需项目。进一步加强监督检查，着力解决项目实施管理中存在的问题，加快项目实施进度。三是强化项目前期工作。配合国家和省上有关部门加快湖北十堰至天水高速公路陇南段、成县至武都高速公路、兰海高速公路渭源至武都段等重大项目前期工作进度，力争尽快开工建设。抓紧做好平凉至天水铁路武都延伸段、成州支线机场前期工作，力争早日启动实施。市县区财政将增加项目前期经费投入，为项目前期工作提供有力保障。四是加大项目争取力度。认真研究国家扩大内需、促进西部地区和甘肃经济社会发展等政策，找准与我市发展的接合点，筛选、论证、申报一批项目，积极争取国家、省上各类投资。抓紧做好已上报1747项争取中央投资项目的衔接工作，重点做好有明确支持意向的10个方面80多个项目的衔接、争取工作。抓好项目储备，不断充实项目库，努力解决后续大项目储备不足的问题。五是提高项目管理水平。通过培训、引进、选调等多种办法，充实项目论证、实施和管理人才队伍。切实改变重项目争取，轻项目管理的状况，加强各类项目特别是中央投资项目的管理，完善管理办法，规范项目审批、核准、备案制度，强化项目稽查，对重点项目实行全过程跟踪督查，确保各类项目发挥效益。六是加大招商引资力度。建立健全工作机制和激励机制，创新招商方式，拓宽招商渠道，加强与友好城市和区域协作城市的经济合作，努力扩大招商引资规模。加强招商引资项目的跟踪服务，切实提高项目合同的履约率和资金到位率，力争全年签约招商引资项目150项以上，签约资金90亿元以上，到位资金40亿元以上，项目履约率达到80%。

三、着力加快工业发展，提升工业质量效益。全面贯彻落实市委二届六次全委扩大会议确定的工业发展指导思想、奋斗目标、工作重点和重大措施，在工业项目招商引资、园区建设、提升质量效益等方面采取更有力的工作措施，加快工业发展，增强工业对经济增长、财政增收的支撑作用。一是加快工业项目建设。尽快出台工业项目招商引资办法，以招商引资推动工业项目建设。着力解决工业融资、工业用地等难点问题，确保38个市列重点项目早日建成投产。二是加快工业园区建设。做好全市工业园区集中区建设布局总体规划，重点抓好西成经济开发区清理整顿和扩区规划编制，尽快完成徽县循环经济工业集中区总体规划，争取通过省发改委审批，尽早启动实施。三是提升工业质量效益。扎实开展科技创新年和质量效益年活动，强化技术引进，提高企业自主创新能力、经营管理水平、产品质量和经济效益。推进矿产资源整合，积极实施重点产业振兴规划，改造提升传统产业，不断延伸产业链条，发展高附加值产品，壮大特色优势产业，推动结构调整和产业升级。鼓励实力较强的企业成立研发机构，实施科技研发项目。加强与大专院校、科研院所的联合协作，引进技术，培养人才。新上项目要采用新技术、新工艺、新材料、新装备。抓好品牌培育，争创名牌产品。四是推进节能降耗。严格执行产业政策，淘汰小水泥、小硅铁等高耗能、高污染产能。加强对高耗能行业和企业的监测，把各项节能目标措施落到实处。抢抓国家支持节能减排、省上实施循环经济规划的机遇，积极论证和争取项目资金，力争在发展循环经济上取得新进展。五是精心组织好工业生产。进一步强化对工业经济运行态势的监测分析，密切关注重点工业产品价格变化和市场走势，及时研究解决工业经济运行中存在的困难和问题。加强对企业的扶持和服务，认真落实国家和省上扶持工业特别是中小企业的各项政策措施，管好用好市级财政扶持工业发展基金，建立中小企业贷款担保基金，努力解决中小企业融资难的问题。支持企业加强市场营销，多方开辟工业产品销售渠道，千方百计提高工业效益，确保全市工业经济平稳较快发展。

四、加快发展特色农业产业，促进农民收入持续增长。坚持农业的基础地位不动摇，进一步加大对“三农”的投入力度，确保粮食稳产增产，加快发展特色农业产业，狠抓劳务输转，促进农民增收。一是全面落实各项强农惠农政策。大力推行惠农财政补贴“一册明、一折统”发放管理办法，确保各类补贴及时足额发放到位。二是加快特色农业产业发展。进一步完善全市特色农业产业发展总体规划，坚持分类指导，推进核桃、花椒、油橄榄、中药材、草食畜、蔬菜、马铃薯、苹果等已经形成规模的产业由大变强，提质增效；发展潜力较大的茶叶、水产养殖、食用菌、蚕桑等产业由小变大，在扩大规模的同时提高质量效益，把特色产品做特做优。加快农产品加工流通龙头企业建设，新建农产品生产加工企业20户。大力发展农民专业合作经济组织，继续做好农产品注册和认证工作。三是大力推广农业科技。抓好农业新品种、新技术、新机械的引进、示范和推广，推进种植业、养殖业结构调整，提高农产品品质，降低农业成本，减少农业污染。深入开展农业科技下乡活动，开展多层次、多渠道、多形式的技术培训，培训农民6.8万人。完成全膜双垄沟播技术面积35万亩。四是加强农业基础建设。加快农田水利建设，年内新修梯田20万亩，发展有效灌溉面积3.6万亩、集雨节灌4万亩、节水灌溉4万亩。继续抓好水土保持工作，实施小流域综合治理410平方公里。坚持抓好退耕还林、天然林保护、生态修复、公益林和自然保护区建设。大力推进太阳灶、沼气池等清洁能源和“一池三改”配套建设。五是加快扶贫开发。进一步加大扶贫开发力度，注重扶贫开发与农村低保“两项制度”的有效衔接，摸清“低保户”和 “扶贫户”的底子，建档立卡，动态管理，做到“应保尽保”、“应扶尽扶”。继续推进整村整片整流域开发，改善贫困地区基础设施和生产生活条件，加大产业扶贫、科技扶贫力度。创新工作机制，整合培训项目，提高劳务输转质量，全年完成农民工技能培训13万人，输转劳务67万人，创收52亿元。做好2011—2020年扶贫开发纲要编制工作，为下一个十年扶贫开发工作打好基础。六是抓好新农村建设示范点创建工作。结合灾后恢复重建，高质量抓好省、市、县区新农村示范点建设，力争在特色产业发展、危房改造、基础设施建设、村容村貌整治等方面取得新进展，建成一批新农村建设示范村。努力争取在有条件的地方开展城乡一体化试点工作。

五、加快交通等基础设施建设，着力改善发展条件。采取更加有力的工作措施，重点解决征地拆迁难度大、配套资金落实困难、技术和施工力量不足等突出问题，力争在交通建设上实现新突破。优化建设环境，全力做好协调服务，狠抓项目进度质量，确保10条国省干线公路改建任务全面完成。继续实施乡村“畅通工程”和“通达工程”，完成通乡油路61条1265公里，努力实现乡乡通油路。开工建设东江和钟楼滩2个一级客运站，8县县城二、三级客运站，新建16个重点乡镇三级汽车站，提升客货运输水平。深化农村公路养护体制改革，全面开展日常养护工作。加快电网建设，抓好临江330千伏送变电工程和成县黄渚、王磨、武都东江等110千伏送变电工程建设，确保项目按期完成投运；积极做好西部农网完善工程项目投资争取工作，新开工建设一批35千伏及以下农村电网项目，进一步提高农村电网供电质量。加强通信建设，实施电信、移动信息化建设合作项目。

六、强化城镇建设管理，大力提升城镇化水平。全面贯彻落实全市城市建设现场会议精神，抢抓国家、省上促进中小城市和小城镇建设的机遇，努力实现城镇建设新突破。进一步加强城市规划的编制和实施工作，加快城市规划修编进度，年内完成陇南市区、各县县城以及哈达铺等20个重点镇的总体规划、控制性详规编修或编制工作。武都作为市委、市政府所在地，要发挥区位相对优势和铁路、高速公路枢纽后发优势，实施以新带旧战略，加快市级行政中心、商贸物流中心和交通枢纽中心建设步伐。加大东江、钟楼滩新区开发建设力度，推进旧城区改造，抓紧实施长江大道东江段和钟楼滩段、市区白龙江堤防、北峪河大桥、南长江大道、城姚路大桥、南北两山绿化等建设项目，充分发挥有山、有水、气候好的优势，努力建设山水园林城市。加快八县县城建设，重点抓好城市道路、供排水、环卫设施、社会服务设施和污水处理等项目建设。制定优惠政策，鼓励农村有条件的群众向城镇转移。做好土地储备，盘活存量土地，提高土地利用效率。加大招商引资力度，引进社会资本、民营资本和外资投入城市建设。做好县乡土地利用总体规划修编工作，加强城镇建设特别是各类建设用地管理，严格执行土地征收征用政策，规范土地征用，坚持依法征地、依法拆迁、和谐拆迁。下大气力加强城市管理，使城市面貌有新变化。

七、加快发展社会事业，坚持办好改善民生的实事。抢抓国家和省上加大对教育、科技、卫生、文化、体育、社会保障等方面投入的机遇，加快社会事业发展。坚持优先发展教育事业，深入研究全市教育发展的重大问题，开展教育改革年和教育质量提高年活动，着力解决教育发展布局不合理、教育机制不活、师资力量薄弱、教育质量不高等广大群众关心的问题。实施好科技项目，培育科技创新和科技示范型企业，深入推进科技特派员创业行动，抓好农民科技培训。加快发展卫生事业，抓好乡镇、村和社区卫生服务机构建设，加快建立覆盖城乡的卫生服务体系；实施好公共卫生项目，加强疾病防控、卫生执法监督，抓好食品药品安全监管。严格执行人口和计划生育各项政策法规，坚持统筹解决人口问题，全面落实各项优先优惠政策，进一步抓好流动人口计划生育管理与服务，加大对出生人口性别比偏高问题的治理力度，稳定低生育水平。抓好第六次全国人口普查。开工建设市博物馆、图书馆、文化馆，加快乡镇综合文化站、农家书屋工程建设进度，整合资源，建设文化大院，继续抓好非物质文化遗产的挖掘保护，建立健全文艺精品创作激励机制；组建广电网络公司，力争年内完成广电网络整合工作，建设广电大厦，全面完成广播电视村村通建设任务。加强城乡体育设施建设，广泛深入开展全民健身活动。抓好防灾减灾项目建设，提高防灾减灾能力。坚持实施积极的就业政策，落实扶持政策，进一步加大就业援助力度，尽最大努力解决普通高校毕业生、军转干部、复转军人和就业困难群体的就业问题，千方百计为下岗失业职工、残疾人家庭、城市零就业家庭等提供救助和服务。进一步建立健全村干部和失地农民养老保障制度，全面推进城镇居民基本医疗保险制度建设，完善未参保集体企业参加养老保险、关闭破产企业退休人员参加城镇职工基本医疗保险制度，努力扩大社会保障覆盖面，不断提高社会保险统筹层次。推进城乡社会救助体系建设，继续做好城乡低保、农村五保供养、临时救助、医疗救助工作。

今年我们将继续办好改善民生的13件实事。新修通村等级公路219条1058公里，新建便民桥100座；解决10万农村人口饮水不安全问题；实施扶贫整村推进100个，减少贫困人口5万人；新建农村沼气池1.5万户，为3万户农民配备太阳灶；通过建设廉租住房、经济适用房、发放住房补贴等办法，解决5500户城市低收入家庭住房困难；安排社会就业1.2万人、高校毕业生就业2000人，解决200所边远乡村学校教师缺编问题；加大地方财政投入，提高城镇职工基本医疗保险标准，用人单位缴费比例提高到工资总额的6%，住院最高支付限额由2万元提高到4万元，实行大病医疗补助；提高新型农村合作医疗筹资水平，补助标准从80元提高到120元，达到每人每年150元，相应提高报销标准；为全市78所乡镇卫生院配备救护车，增强乡镇卫生院的急救能力；新建“五保家园”150所，提高“五保户”集中供养率；加强食品安全监管，改造200个食品加工小作坊；开展城乡环境卫生综合整治，加强环卫设施和公益设施建设，年内力争使九县区城区生活垃圾填埋场建成投用，切实改变城区脏乱差；对国道、省道干线公路39个以路为市、占道经营的马路市场进行整顿和扩建，确保交通安全畅通。

八、大力发展第三产业，做好财税金融工作。认真贯彻国务院《关于加快发展旅游业的意见》，推动旅游产业发展，全面完成十大名牌景区和十大重点景区（点）修建性详规编制，加强景区道路、宾馆饭店等旅游基础设施建设，加强旅游质量规范管理和旅游从业人员培训，开发旅游纪念品、工艺品和特色旅游商品，开展旅游宣传推介，多方开辟客源市场，力争全年旅游人数、旅游综合收入比上年有较大增长。继续抓好“万村千乡”市场工程和“新网工程”，加快40个续建和新建市场项目建设。鼓励城乡居民扩大住房、汽车、通信、教育、体育、文化等方面的消费，实施好“家电下乡”、“汽车以旧换新”，扩大城乡消费。加快培育奶花芸豆、核桃、中药材、苹果、大蒜、肉牛等进出口企业和出口产品，扩大外贸进出口。加强地方粮食储备，确保粮食安全。切实加强财政增收节支工作，依法加强税收征管，完善政府非税收入征缴制度。加强重点行业和潜力税源、零散税源征管，大力培植和挖潜新兴税源。将国有资源、行政事业单位国有资产经营收益全部纳入财政综合预算，实行收支两条线管理。优化财政支出结构，压缩一般性支出，保证“三农”、社会事业、灾后重建等重点支出。加强金融保险保障工作，围绕全市灾后恢复重建和经济社会发展重点，扩大信贷投放；加强社会信用体系建设，不断改善金融生态环境，促进银地、银企合作。

九、坚持体制机制创新，推进重点领域改革。推进政府机构改革，全面完成市县区政府机构改革任务。继续推进国有企业改革，做好已改制企业的后续完善工作，解决好遗留问题。全面推进集体林权制度改革，完成兽医体制、基层农技推广体系、农产品质量安全监管体系、农业行政执法体系等改革任务，开展农村集体土地流转试点工作。推进医药卫生体制改革，进一步完善新型农村合作医疗制度，开展基层医疗机构实行药品零差价销售试点工作，探索公立医院改革。推进财政管理体制改革，加快推进部门预算、国库集中收付、政府采购、行政事业单位国有资产管理改革，继续推进非税收入管理改革，加快建立财政支出追踪问效机制。加快实施以岗位设置管理为重点的事业单位改革，严格执行事业单位聘用制度。深化科技、文化体制改革，增强社会事业发展活力。深化农村信用社改革，积极发展面向中小企业和农村的村镇银行、小额贷款公司等新型金融机构。推进投融资体制改革，发挥城投公司、中小企业担保中心等融资机构的作用。强化资本运作，争取有条件的企业上市融资。

十、全力维护社会稳定，促进社会和谐。坚持稳定是发展的前提，是“第一责任”的原则，扎实做好维护社会稳定工作。加强群众来信来访办理工作，畅通群众诉求表达渠道，坚持领导干部接待群众来访、包案处理信访问题、带案下访等制度；进一步规范信访秩序，教育引导群众依法、有序表达合理诉求，从源头上预防集体上访、越级上访、非正常上访。深入开展矛盾纠纷排查调处，建立经常性的矛盾纠纷排查调处和预防机制，努力把矛盾纠纷化解在基层，化解在萌芽状态。强化基础工作，加大普法力度，深入开展人民调解、安置帮教和社区矫正、法律援助工作，提高乡镇、社区化解社会矛盾的能力。深入推进社会治安防控体系建设和“平安陇南”创建，强化社会治安综合治理，严厉打击各类违法犯罪，不断增强人民群众的安全感。加强应急管理体系建设，完善应急预案体系，提高应对和处置突发公共事件、保障公共安全的能力。严格落实安全生产责任制，加大安全生产投入，加强消防工作，强化重点领域的安全监管和整治，防止重特大事故发生。全面贯彻落实党的民族宗教政策，加快少数民族乡村经济社会发展，加强民族团结宣传教育，依法加强宗教事务管理。加强民兵预备役建设，支持驻陇南部队建设，坚持开展全民国防教育，深化双拥共建。发展老龄、残疾人和慈善事业，做好老干部工作；支持工会、妇联、共青团等群众团体的工作，维护职工、妇女儿童和青少年的合法权益，促进社会和谐。

加强政府自身建设

各位代表，今年全市灾后恢复重建和发展改革稳定任务十分繁重。我们要奋力开创陇南科学发展新局面，就必须坚持以邓小平理论和“三个代表”重要思想为指导，深入学习实践科学发展观，认真学习国家的政策法规，学习经济政治、文化科技、财税金融、公共管理等各方面知识，切实加强政府自身建设，全面履行经济调节、市场监管、公共服务、社会管理职能，不断提高依法执政、科学执政、民主执政的能力和水平。

一是加强行政监督，努力建设责任政府。始终坚持对人民负责、对组织负责、对社会负责，做到有权必有责，用权受监督。认真贯彻国家方针政策，坚决执行省委、省政府和市委的决策部署，自觉接受人大依法监督、政协民主监督和公众舆论监督，认真执行市人民代表大会及其常务委员会的决定、决议，坚持重大事项报告制度，虚心听取人大代表、政协委员、社会各界人士的批评、意见和建议，进一步提高人大代表建议、政协委员提案办理质量。坚持从严治政，严格管理，强化目标责任管理和绩效考核，完善行政问责制度，加大责任追究力度，对不负责、失职渎职，造成重大损失或恶劣影响的，要严肃进行责任追究，促使广大公务员认真履行职责，自觉做好工作。

二是提高依法行政水平，努力建设法治政府。深入实施依法行政纲要，强化法治观念，把依法行政贯穿到政府工作的各个领域、各个环节，努力做到合法行政、程序正当、高效便民、诚实守信、权责统一。严格按照法定权限和程序履行职责，既不能失职不作为，也不能越权乱作为。完善政府规章制度，依法解决经济社会发展中的各种利益矛盾。建立健全依法行政各项配套制度，探索相对集中行政处罚权和综合行政执法试点工作。加强行政执法队伍建设，强化对执法人员的管理和监督，进一步规范行政执法行为，完善行政执法程序。加强行政复议和行政应诉工作，不断提高依法处理经济社会事务的能力。进一步推进政务公开，健全完善信息公开和新闻发布制度，发展电子政务，完善公众信息平台，保障公民对公共事务管理的知情权、参与权、表达权、监督权。

三是切实改进工作作风，努力建设效能政府。坚持求真务实，把提高执行力和抓落实的能力，作为各级政府作风建设的落脚点，进一步健全完善抓落实的相关制度，用制度促进和保障工作落实。继续实行领导干部包抓项目、包抓产业制度和联系县区、乡村、企业、学校及创办示范点的制度；坚持重点工作、重点任务定期督查制度，改进督查方法，讲求实际效果；市政府每季度召开一次重大项目、重点工作进度分析调度会，通报工作进展情况。建立严格的会议、文件审批制度，继续精简会议、文件，改进文风、会风，把更多时间、精力用到抓工作落实上来。

四是着力优化发展环境，努力建设服务政府。各级政府要切实履行优化发展环境的职责，以促进政府职能转变、强化公共服务、提高服务效率和服务质量为着力点，扎实开展“优化发展环境，塑造良好形象”活动，把解决突出问题与构建长效机制结合起来，既要严肃查处破坏环境的人和事，又要注重制度建设，规范办事程序，规范权力运行，进一步健全落实“首问责任”、“限时办结”、“一站式”服务、行风评议等制度。通过开展阶段性集中教育和整治活动，使发展环境有明显变化，服务发展、服务项目建设的意识明显增强，服务质量、服务效能明显提高，基层矛盾纠纷得到有效化解，人民群众和市场主体对软环境的满意度有较大提升。

五是扎实推进廉政建设，努力建设廉洁政府。认真落实廉政建设“一岗双责”责任制，做到廉政建设与中心工作同部署、同落实、同检查、同考核。加强廉洁从政教育，筑牢反腐倡廉思想道德防线。加大查办违纪违法案件工作力度，严肃查处各类违纪违法案件。加强执法和行政效能监察，健全权力运行机制，认真落实党政干部问责制、廉政承诺制、行政执法责任制等制度，完善党政主要领导干部和国有企业领导人员经济责任审计，加强对财政资金、灾后重建资金、重大投资项目资金的监管。推进反腐倡廉制度创新，坚持用制度管权、管事、管人，严格落实政府投资监管、国有资产监管、非经营性资产管理以及建设工程招投标、经营性土地使用权出让、政府采购、产权交易等各项制度，推进惩防体系建设。坚决纠正损害群众利益的不正之风，维护广大群众的权益。大力倡导艰苦奋斗，厉行勤俭节约，把有限资金和资源更多地用在发展经济、改善民生上。

各位代表，站在历史的新起点，面对人民的新期待，我们深感责任重大、使命光荣。展望陇南科学发展的美好未来，我们有信心、有决心把陇南人民的事情办好，把陇南的发展不断推向前进。让我们在省委、省政府和市委的坚强领导下，在市人大、市政协的有力监督和全市人民的大力支持下，同心同德，万众一心，艰苦奋斗，扎实工作，为全面完成灾后恢复重建任务，推动全市经济社会又好又快发展而努力奋斗！
